# Visual training for the treatment of retinal photoreceptor inner and outer segment connection (IS/OS) fracture: A case report

**DOI:** 10.1097/MD.0000000000038109

**Published:** 2024-05-31

**Authors:** Peng-Fei Jiang, Shan Xu, Qing Chen, Bing-Xin Pan

**Affiliations:** a Quzhou Hospital of Zhejiang Medical and Health Group, Quzhou, China.

**Keywords:** photoreceptor inner and outer segment connections, retina, visual training

## Abstract

**Background:**

The inner segments and outer segments (IS/OS) of the retinal photoreceptors are the areas that receive light signals and are the most initial sites for generating visual impulses, and the integrity of the IS/OS has a direct impact on visual sensitivity.

**Methods:**

We performed OCT on a 6-year-old child with vision loss and found that the cause of his vision loss was a retinal IS/OS fracture, and the child underwent some treatments to improve microcirculation and nourish the retina at a higher-level hospital, but his vision never improved. Our examination of this child revealed that this child not only had decreased visual acuity, but also hypermetropia, but his near stereopsis was normal. The symptoms were similar to those of amblyopia, so we tried to use visual training as a treatment.

**Results:**

First, 6 sessions of fine visual stimulation were given, followed by 3 sessions of accommodation training, and we followed the 4 stages of accommodation training: perception of accommodation, amplitude of accommodation, sensitivity of accommodation, and autonomic accommodation. After 9 consecutive visual training sessions, the child’s visual acuity was stabilized at 0.6, and then we added eye movement training, and after the child’s visual acuity was improved to 0.7, we suppressed the visual acuity of the left eye to 0.6, so as to make the visual acuity of both eyes similar, which would promote the establishment of binocular stereo vision, and then we carried out 9 more visual training sessions, and the patient’s visual acuity was stabilized at 0.8 gradually. OCT review showed that the child’s retinal IS/OS fracture was basically closed.

**Conclusion subsections:**

In conclusion, our study found that visual training can restore visual acuity in children with monocular IS/OS fracture and also promote repair of IS/OS fracture, which increases our understanding and knowledge of the treatment of retinal IS/OS fracture, and this case may provide some lessons for the treatment of retinal IS/OS fracture in children. We hope to have more samples of retinal IS/OS fracture in the future to evaluate the efficacy of visual training for retinal IS/OS fracture.

## 1. Rationale

Photoreceptors are long and narrow cells, belonging to the nerve terminal cells. Photoreceptors are rich in organelles with cellular metabolic activity, and their active metabolism and renewal cause the cells to be particularly sensitive to injury. Park et al^[1]^ concluded that the incomplete IS/OS of the photoreceptor cells may be caused by reversible photoreceptor outer segment alteration and irreversible photoreceptor death by examining the retinal shock cases. IS/OS fracture are very difficult to heal spontaneously, even with the use of treatments such as nutrient retina to improve the microcirculation. We treated a 6-year-old child with retinal IS/OS dissection and used visual training to restore his visual acuity, and the retinal IS/OS dissection was basically healed.

## 2. Patient concerns

A 6-year-old child’s vision in the right eye had decreased for several days without any obvious reason. No history of hypertension, diabetes mellitus, surgery, trauma, drug allergy or long-term drug use. Ophthalmologic examination: visual acuity: 0.3/1.0, intraocular pressure: 15/16 mm Hg, computerized optometry: right + 0.50/−1.50*88, left + 0.25/−0.25*84. Pupils of both eyes had normal light reflexes, and refractive interstices were clear. Funduscopic examination: the borders of the optic discs of both eyes were clear, the color was normal, the retinal blood vessels were normal, and a round dark red area with clear borders was seen in the macular area, with the central concave reflection disappearing. OCT (Spetraslis-OCT model optical coherence tomography scanner manufactured by Heidelberg, Germany, the scanning area was 9.0 mm × 9.0 mm, the scanning mode was Retina 30-degree fundus 9 mm, the scanning length, the scanning depth was 1.0 mm, and the scanning depth was 1.0 mm, and the scanning depth was 1.0 mm. (Scan length, scan depth 1.9 mm, axial resolution 3.9 μm, transverse resolution 5.9 μm).

## 3. Diagnoses

**Figure 1:**
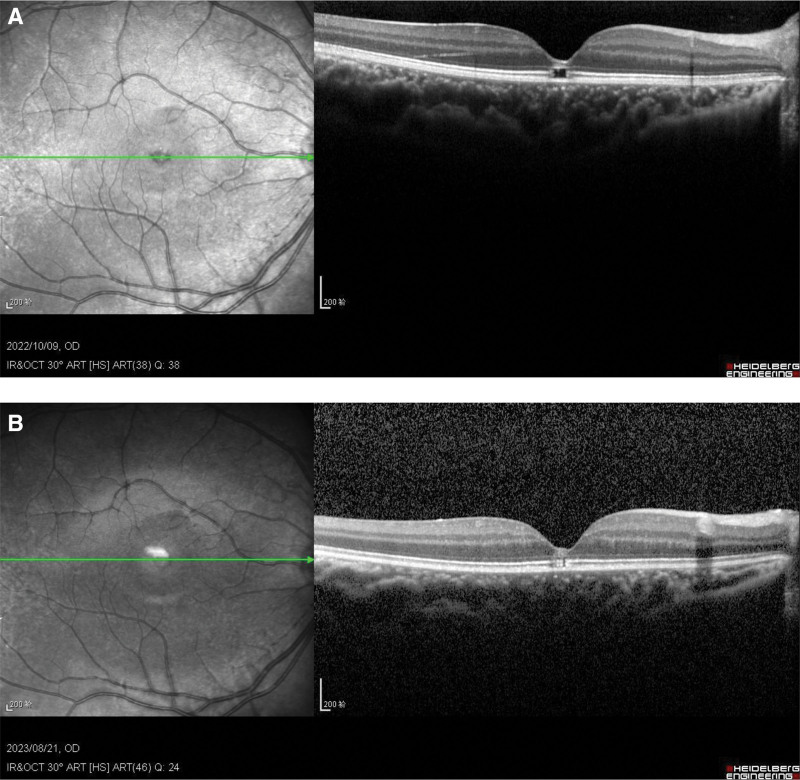
(A) October 9, 2022, retinal OCT showing IS/OS fracture. (B) August 31, 2023, retinal OCT showing substantial closure of the IS/OS fracture. IS/OS = inner segments and outer segments

The diagnosis was a fracture in the IS/OS of the photoreceptors in the right eye (Fig. 1A).

## 4. Interventions

Since then the child had been treated at a higher hospital for improving retinal microcirculation and nourishing the retina, but the treatment effect was not obvious.

The child came back to our hospital on April 18, 2023, and we performed a detailed examination of the visual function with the following results:

Atropine prescription: od + 2.00/−1.00 × 92-0.3, os + 1.75-1.0.
Prescription for lenses: OD + 0.50/−1.00 × 92-0.3, OS + 0.50-1.0.
Naked eye visual acuity (distance): OD 0.3, OS 1.0.
Assembled near point: 4 cm.
Masking experiment: far and near orthoptic.
Adjustment amplitude (advancing method): OD 14D (0.4 visual acuity), OS 14D (0.8 visual acuity).
WORTH 4 points: 4 lights both near and far.
Stereopsis (Bernell red and green book): far: none, near: 100 seconds.

Negative relative regulation, positive relative regulation could not be detected because the difference in visual acuity between the left and right eyes was too large.

The child’s right eye visual acuity decreased significantly, and at the same time, there was hyperexcitability of the right eye, blurred vision, but there was a more normal near stereopsis. Therefore, our idea of visual training is as follows: firstly, give enough fine visual stimulation training to stimulate the excitability of visual nerve cells, according to the treatment of amblyopia; after the visual acuity fracture through 0.5, increase the training of the adjustment function, according to the 4 stages of the regulation of perception, the magnitude of the regulation, the regulation of sensitivity, and the autonomy of the regulation of the 4 stages of the hierarchy; after the child’s visual acuity is further improved to 0.6, increase the training of the ocular motility from After the visual acuity was further improved to 0.6, the training of eye movement ability was increased, from gaze to sweep to follow; The left eye was mainly covered in the early stage, and after the visual acuity was improved to 0.7, it was changed to the left eyeglass lens to press and paste 0.6 specification suppression film, to promote the formation of binocular distance stereo. This study was approved by the Ethics Committee of Zhejiang Medical and Health Group Quzhou Hospital. The guardian of the child has signed a patient consent.

## 5. Outcomes

The child started visual training on May 16, 2023, and was admitted to the hospital once a week for training. On August 31, 2023, the child was reexamined and the right eye was wearing a lens of 0.8 and the left eye was wearing a lens of 1.0. At present, the child’s visual acuity was stable, and the visual training was changed to once every 2 weeks. The visual acuity in the right eye was 0.8 on August 31, 2023, and the left eye was 1.0. The OCT showed that the retinal IS/OS fracture had basically been closed (Fig. 1B).

## 6. Lessons subsections

The inner and outer segments of the retinal photoreceptors are the areas that receive light signals and are the most initial sites for generating visual impulses, and the integrity of the IS/OS directly affects visual sensitivity.^[2]^ Thus IS/OS fracture can lead to significant vision loss.^[3]^ A retrospective, comparative, consecutive case study from the Department of Ophthalmology at the University of California, San Diego included 62 eyes of 38 patients with diabetic macular edema undergoing SD-OCT imaging and showed a significant correlation between the percentage of disruption of the photoreceptor inner-segment-outer-segment connection and visual acuity in diabetic macular edema (*P* = .0312).^[4]^ Researcher Liu et al^[5]^ observed the effect of localized photoreceptor fracture in the macular central recess on visual acuity, and included 31 eyes (22 eyes with photoreceptor whole-layer fracture and 9 eyes with photoreceptor outer-segment fracture) of 31 patients with IS/OS fracture, and 30 normal individuals matched for age and refraction as a control group, and found that localized photoreceptor fracture in the macular central recess could cause visual acuity loss, and the greater the extent of the fracture, the more pronounced the decrease in visual acuity.

When performing fundus examination on patients with IS/OS fracture, it is also very easy to miss the diagnosis because the change of IS/OS fracture at the macular center recess is extremely subtle, and currently OCT can observe IS/OS fracture more clearly, so OCT is a better examination method to diagnose IS/OS fracture.

There are more causes of IS/OS damage, which can be combined with macular diseases such as idiopathic macular lacunae, macular splitting in high myopia, idiopathic macular anterior membranes, and is also seen after macular lacunae surgery, and trauma, light stimulation, and thermal stimulation can also lead to IS/OS fracture.^[6,7]^ Hyperthermia can also cause monocular or binocular IS/OS fracture, visual acuity can be significantly reduced, visual evoked potentials suggest damage to the visual conduction pathway in both eyes. Researcher Lu^[8]^ reported 2 cases of posthyperthermia IS/OS fracture in patients who were treated with vasodilator medication and vitamins and other nutritive nerve medication, and there was no significant improvement in their visual acuity. When the IS/OS is broken, the morphology of photoreceptor cells is damaged, and such destruction of morphology can generally be considered as death of photoreceptor cells.

Although IS/OS fracture can be detected by careful observation of OCT images, there is no consensus on the treatment of IS/OS fracture. Because of the low prevalence of IS/OS fracture, they have been reported less frequently. Previous reports have found that treating IS/OS fracture with nutritive nerve and circulation-improving drugs such as multivitamins and Ginkgo biloba extract injections did not correct vision, and OCT review after 1 year of treatment revealed that IS/OS fracture had disappeared, but the impaired vision still could not be restored.^[9]^ This suggests that drug treatment may have limited efficacy for IS/OS fracture.

We found that the clinical manifestations of IS/OS fracture in this child were not only visual acuity loss, but also reduced accommodation function, but his near stereopsis was normal. The symptoms were similar to those of amblyopia, so we tried to use visual training as a treatment. First, 6 sessions of fine visual stimulation training were given, and then 3 sessions of accommodation training were given. We followed the 4 stages of accommodation training, namely, accommodation perception, accommodation amplitude, accommodation sensitivity, and autonomic accommodation, in order to progress step by step. After 9 consecutive visual training sessions, the child’s visual acuity was stabilized at 0.6, and we added oculomotor training. After the child’s visual acuity was improved to 0.7, we suppressed the visual acuity of the left eye to 0.6, so as to make the visual acuity of both eyes similar, which would promote the establishment of binocular stereoscopic vision, and then we carried out 9 more visual training sessions, and the patient’s visual acuity was stabilized at 0.8 gradually.

It should be noted that because IS/OS fractures have a great impact on photoreceptors, the child’s cooperation was poor during the first visual training, but as the number of training sessions increased, the child’s visual acuity improved significantly, and the child’s cooperation with the training gradually improved.

There was no effective treatment for retinal IS/OS fracture before this case, and this case may provide a reference for the treatment of retinal IS/OS fracture in children. We hope to have more samples of retinal IS/OS fracture in the future to evaluate the efficacy of visual training for retinal IS/OS fracture. The younger age of the children and the ability of the retina to repair itself may have led to a more pronounced effect of visual training on visual acuity.

This study also has certain limitations, mainly because we are not aware of the cause of retinal IS/OS fracture in the patient, which to some extent limits the generalizability of the results of this study.

## Acknowledgments

The authors sincerely thank the patients for participation in the present study.

## Author contributions

**Data curation:** Peng-Fei Jiang, Shan Xu, Qing Chen.

**Writing – original draft:** Peng-Fei Jiang.

**Writing – review & editing:** Bing-Xin Pan.
